# Caring, compassion and competence in healthcare

**DOI:** 10.4102/hsag.v27i0.2133

**Published:** 2022-12-12

**Authors:** Nompumelelo Ntshingila

**Affiliations:** 1 Department of Nursing, Faculty of Health Sciences, University of Johannesburg, Johannesburg, South Africa

The theme for this section focused on caring, compassion and competence in healthcare. Caring as a phenomenon and as a science is about human beings (Karlsson & Pennbrant 2020). As stated by Watson (2022), caring as a science is grounded in nursing scholarship and is also relevant to other disciplines in the academia, such as feminist studies, peace studies, education, ethics and human service fields, for example, social work and healthcare professions generally.

Various authors have termed compassion as the fundamental concept in healthcare (Papadopoulos & Ali 2016; Pehlivan & Güner 2020). Following a systematic literature review conducted by Perez-Bret, Altisent and Rocafort (2016), compassion is defined as ‘the sensitivity shown to understand another person’s suffering, combined with a willingness to help and to promote the wellbeing of that person and to find a solution to their situation’. To be compassionate requires understanding the pain and experiences of others. The concept of compassion comprises respect and awareness of others. Bradshaw (2011) confirms that compassionate care as a concept dates back to the time of Florence Nightingale and is associated mainly with nursing as a profession. Compassionate care is instead expressed in actions rather than words. The actions needed are firm touch, gentle, courteous manner and kindness (Bradshaw 2011). Furthermore, compassionate care is said to entail a set of four attributes: wisdom, humanity, love and empathy (Su et al. 2020).

Core competence in healthcare is for the healthcare provider to practice skills that meet the needs of the patients using logical thinking (Fukada 2018). Healthcare professionals must be competent providers of compassionate care. Pehlivan and Güner (2020) highlight the benefits and challenges of providing compassionate care in various healthcare environments. The benefits are improving understanding of involving patients and families in care, determining patients’ and families’ needs, using appropriate approaches and improving patient outcomes. The challenges are work environment challenges and individual factors. Babaei and Taleghani (2019) further included sociocultural barriers such as gender and lack of mutual language as barriers to compassionate care.

For the 2022 *Health SA Gesondheid* special collection, several manuscripts were submitted under the theme of caring, compassionate care and competence. This theme had the most submissions. The manuscripts reviewed under this theme ranged from qualitative, quantitative and systematic reviews. The issues addressed pertained to the challenges of providing competent, compassionate care across the healthcare context. These were challenges related to patients, healthcare providers and students. The manuscripts addressed current issues of coronavirus disease 2019 (COVID-19), gender-based violence, post-exposure prophylaxis and mental health challenges in the healthcare context. The studies’ settings were urban and rural contexts in South Africa and Namibia.

It is evident from the submissions that compassionate care and competence is a theme that will generate conversations in the healthcare context and seems to be of significant interest. I am positive that the articles that are published on the theme of caring, compassion and competence will provide an opportunity for further evidence-based research.
